# The Effects of Immediate Versus Delayed Timing of AI Support on EFL Learners’ Reading Comprehension Performance: An Experimental Study

**DOI:** 10.3390/jintelligence14090199

**Published:** 2026-09-01

**Authors:** Mohammad Hamad Al-khresheh, Suheyla Demirkol Orak, Wael Alharbi, Mayez Almayez

**Affiliations:** 1 Humanities and Social Research Center, Northern Border University, Arar 73213, Saudi Arabia; 2 Department of Foreign Languages, Faculty of Education, Fırat Unıversity, Elazığ 23100, Türkiye; 3 English Language Department, Yanbu English Language and Preparatory Year Institute, Royal Commission for Jubail and Yanbu, Yanbu Industrial City 46452, Saudi Arabia; 4 English Language Department, College of Arts, University of Ha’il, Ha’il 55473, Saudi Arabia

**Keywords:** artificial intelligence, EFL reading comprehension, instructional scaffolding, reading support timing, second language reading, AI-assisted learning

## Abstract

The growing integration of artificial intelligence (AI) into second-language reading instruction has intensified debate regarding whether AI-mediated support facilitates comprehension or reduces the cognitive effort required for independent meaning construction. Although previous research has reported generally positive effects of AI-assisted reading, limited attention has been directed toward the timing of support during the reading process itself. The present study examined whether immediate versus delayed AI support produced different effects on EFL learners’ reading comprehension performance. A pretest–posttest experimental design was employed, involving 90 undergraduate EFL learners, randomly assigned to three instructional conditions: Immediate AI Support, Delayed AI Support, and No-Support control. The intervention was conducted over a ten-week period using AI-assisted reading tasks administered under controlled instructional conditions. Reading comprehension performance was measured using validated pre- and post-test instruments that assessed literal, inferential, and global comprehension. Psychometric validation procedures incorporating Classical Test Theory and Item Response Theory supported the instrument’s reliability and measurement adequacy. One-way ANOVA revealed statistically significant differences across the three instructional conditions, F(2, 87) = 18.679, *p* < .001, η^2^ = 0.301. Post hoc comparisons demonstrated that learners receiving delayed AI support achieved significantly higher post-test reading comprehension scores than learners in both the immediate support and no support conditions. Learners receiving immediate AI support similarly outperformed the no-support group. The findings indicate that the effects of AI-assisted reading varied according to when explanatory support became available during the reading process. Delayed AI support produced stronger reading comprehension performance than immediate support and unsupported reading, suggesting that support timing constituted an important condition shaping comprehension performance in AI-mediated reading environments.

## 1. Introduction

AI-mediated support in second language reading introduces a fundamental tension: it can facilitate comprehension while reducing the cognitive effort required to construct meaning (Bahari et al., 2023). This tension is particularly consequential in EFL contexts, where reading development depends on learners’ capacity to engage in inferencing, resolve lexical gaps, and integrate textual information with prior knowledge under conditions of limited linguistic resources (Kuperman et al., 2023; Samiei & Ebadi, 2021). When AI-generated explanations are made available during reading, they may assist task completion without requiring sustained engagement with the text, particularly through glossing and real-time feedback mechanisms that reduce processing demands (Ünal, 2023; Wang et al., 2025). When access is postponed, learners are required to attempt interpretation independently before consulting external input, thereby maintaining engagement with effortful processing and meaning construction (Peng et al., 2024; Smith et al., 2021). The distinction concerns the role of effort in comprehension rather than the presence of support itself.

Current research on AI use in language learning has largely been organised around general performance outcomes and forms of scaffolded support rather than the temporal conditions under which assistance is accessed (Fan, 2025; Rad, 2025). Such approaches provide limited insight into how AI alters the conditions under which comprehension is constructed during reading. In many cases, AI is treated as a uniform intervention, with support embedded continuously in the reading process without isolating when learners rely on it (Ortega et al., 2022). As a result, reported gains in comprehension cannot be clearly attributed to changes in cognitive processing; they may reflect the availability of immediate explanations rather than differences in how meaning is developed (Çelik et al., 2024; Odo, 2025). The timing of support, consequently, remains undertheorised and insufficiently examined in controlled experimental research.

The absence of this distinction constrains both theoretical accounts of AI-assisted comprehension and pedagogical decisions regarding its integration into reading tasks. When support provides immediate access to explanations, it may reduce the need for inferential processing and weaken engagement with the text, particularly when learners rely on externally generated input (Silor & Silor, 2025). In contrast, conditions that require learners to engage with the text before accessing support are more likely to sustain the cognitive effort necessary for comprehension (Filderman et al., 2022; Castro-Alonso et al., 2021). Determining which of these conditions yields stronger learning outcomes requires treating support timing as an experimental variable rather than treating AI use as a general instructional feature.

The present study addresses this issue through a controlled comparison of three conditions: immediate AI support during reading, delayed AI support following reading, and a no-AI control condition. Using a pretest–posttest design, the study examines whether differences in the timing of AI support affect EFL learners’ reading comprehension performance. The central objective is to determine whether the timing of AI assistance affects the extent to which learners engage in independent processing, and whether these differences are reflected in post-test comprehension outcomes.

## 2. Literature Review

### 2.1. Reading Comprehension and Cognitive Effort in EFL Contexts

Reading comprehension in a second language is widely conceptualised as a process of meaning construction in which readers integrate textual input with prior knowledge to form a coherent mental representation. Within construction–integration frameworks, comprehension develops through successive stages, progressing from lexical decoding to the formation of a situation model that reflects the text’s intended meaning (Hopp, 2022). This process requires continuous evaluation and revision as readers connect propositions and resolve inconsistencies across the unfolding discourse (Yapp et al., 2021). Reiber-Kuijpers et al. (2021) further note that digital reading environments intensify this process by increasing demands on integration and coherence building.

The construction of meaning depends on the coordination of several interrelated processes. Lexical processing provides the foundation for comprehension, with vocabulary knowledge and morphological awareness determining the extent to which readers can establish a stable text base (Teng & Zhang, 2024). Empirical work has shown that vocabulary breadth functions as a primary predictor of reading comprehension in EFL contexts (Brooks et al., 2021). In addition, incidental vocabulary learning contributes to the gradual strengthening of lexical representations that support comprehension over time (Webb et al., 2023). Inferential processing extends beyond this level by linking explicit textual information with implicit meaning, allowing readers to construct coherence where connections are not directly specified (Samiei & Ebadi, 2021). Çelik et al. (2024) demonstrate that inferential comprehension remains sensitive to the form of support provided during reading, indicating that it is a central component of meaning construction rather than a peripheral skill.

Prior knowledge plays a central role in second language reading by providing the conceptual framework through which textual information is interpreted and integrated, enabling readers to resolve ambiguity and construct coherent representations of meaning (Reiber-Kuijpers et al., 2021). This process becomes more demanding in EFL contexts, where linguistic limitations constrain the efficiency of bottom-up processing and increase reliance on top-down resources. Restricted vocabulary knowledge and slower syntactic processing require readers to engage in controlled, effortful processing, allocating greater attention to decoding and integration (Nahatame, 2021). Evidence from large-scale eye-movement research indicates that second language readers exhibit longer fixation durations and more frequent regressions, reflecting the increased cognitive effort required to maintain coherence under such constraints (Kuperman et al., 2023). Although multilingual processing strategies can partially compensate for these limitations, they do not eliminate the underlying processing burden associated with constructing meaning in a second language (Yuzlu & Dikilitaş, 2022).

This increased processing demand requires readers to use cognitive and metacognitive strategies throughout the reading process. They plan, monitor, and evaluate their understanding, modifying their approach whenever comprehension difficulties arise. Chinpakdee and Gu (2024) found that explicit strategy instruction improves learners’ ability to deal with these demands, particularly during inferential comprehension. Similarly, self-regulated learning frameworks describe reading as an active process in which learners monitor and control their cognitive and metacognitive resources while completing the task (Zhang & Zou, 2024). Recent research has also shown that structured support environments, including AI-mediated scaffolding, can strengthen learners’ strategic engagement by encouraging monitoring and adjustment during reading (Pan et al., 2025). These findings suggest that successful reading comprehension depends not only on learners’ linguistic knowledge but also on their ability to regulate cognitive resources while responding to the demands of the text. Consequently, an important question for reading instruction is whether external support changes how learners engage with these processes during comprehension.

### 2.2. External Support and the Regulation of Cognitive Effort

Instructional support in reading redistributes cognitive effort across the processes involved in comprehension rather than simply making reading tasks easier. Van Nooijen et al. (2024) describe instructional support within cognitive load theory as a way of directing attention, structuring information, and reducing unnecessary processing demands associated with decoding and integration. As unnecessary processing demands are reduced, learners can devote more cognitive resources to understanding the text. Segmentation of textual input introduces pauses that help learners process information incrementally, reducing overload while maintaining engagement with comprehension processes (Liu, 2024). Adaptive scaffolds and analytics-based systems also encourage learners to monitor their understanding, reread difficult sections, and organise information more effectively (Li et al., 2023). In digital reading environments, support is increasingly integrated into the reading process itself, allowing learners to access assistance whenever difficulties arise and influencing how comprehension develops during reading (Rad, 2025).

The effectiveness of instructional support depends on how well it matches both the demands of the reading task and learners’ capabilities. Castro-Alonso et al. (2021) argue that effective instructional design should reduce extraneous cognitive load while preserving the processing required for comprehension. When reading demands exceed learners’ available cognitive resources, well-designed support helps direct attention toward information most relevant for comprehension without removing the need for active engagement with the text (Peng et al., 2024). This effect becomes more pronounced when instructional support combines strategy instruction with background knowledge, reducing the effort required for comprehension while strengthening strategic reading. Filderman et al. (2022) found that interventions targeting core comprehension processes generally produce stronger outcomes than those introducing additional instructional features without a clear functional purpose. Reading tasks should remain sufficiently challenging to sustain active engagement without overwhelming learners’ cognitive capacity (Smith et al., 2021). Mežek et al. (2022) likewise argue that task design should encourage planning, monitoring, and evaluation throughout the reading process.

However, instructional support also introduces an important challenge. The same support that reduces unnecessary processing demands may also reduce the need for learners to work through meaning independently. When support provides direct explanations or divides attention across multiple sources of information, it may limit inferential processing and interfere with the development of a coherent understanding of the text. Skulmowski and Xu (2022) showed that instructional features that are poorly aligned with learners’ processing needs may either overload learners or reduce productive engagement with the text. Similarly, research on digital reading suggests that embedded support tools can assist local comprehension while disrupting broader understanding because learners’ attention is divided across multiple sources of information (Furenes et al., 2021). This issue becomes particularly important in AI-supported reading, where explanations are available immediately whenever learners encounter difficulty. Under these conditions, AI-generated support may complement learners’ own processing or partially replace it, depending on when support is accessed during reading (Rad, 2025). This distinction provides the conceptual basis for examining how the timing of AI support influences cognitive effort and, ultimately, reading comprehension outcomes.

### 2.3. AI-Mediated Support as a Processing Intervention

AI-based tools in second-language reading function as part of the comprehension process itself rather than as external sources of assistance. Yuan (2025) conceptualises AI-supported environments as systems that dynamically adjust text difficulty and provide real-time feedback, allowing support to be integrated into reading as comprehension unfolds. Earlier work on digital glossing similarly demonstrated that annotations influence how readers attend to textual information and process meaning during reading (Lomicka, 1998), a finding later extended to multimodal and contextual glossing environments that influence both lexical access and inferential processing (Ünal, 2023; Ortega et al., 2022). AI-based simplification and rephrasing further modify linguistic input by reducing textual complexity, although their effectiveness depends on learner characteristics and task demands (Çelik et al., 2024; Odo, 2025). These forms of support influence more than comprehension outcomes alone. Fan (2025) found that AI-based scaffolding promotes higher-order reading strategies, particularly among learners with lower working memory capacity, whereas retrieval-based activities increase processing time and strengthen form–meaning connections, indicating deeper cognitive engagement (Wang et al., 2025). From a cognitive load perspective, AI can reduce unnecessary processing demands while preserving the cognitive activity required for comprehension (Bahari et al., 2023). Adaptive scaffolds have also been associated with greater monitoring and self-regulation during reading (Rad, 2025), and interactive AI systems can sustain learners’ engagement throughout reading tasks (Silor & Silor, 2025). The evidence suggests that AI shapes how learners engage with texts by influencing attention, strategy use, monitoring, and cognitive engagement rather than simply making reading easier.

AI-mediated support also reshapes reader agency and critical engagement, introducing interactional dynamics that extend beyond comprehension toward evaluation and regulation. Wei (2023) links continuous AI feedback to the development of self-regulated learning behaviours, particularly in relation to monitoring and strategy adjustment during reading. Dialogic AI systems further transform reading into an interactive process, in which learners engage in questioning and the iterative refinement of interpretations, thereby altering how meaning is negotiated during comprehension (Lin et al., 2025). Thongsan and Anderson (2025) show that such interaction can shift readers toward evaluative engagement, where AI responses are questioned and refined rather than accepted uncritically. This shift requires readers to assess the reliability of AI-generated input, thereby positioning AI as an object of evaluation rather than a source of authority (Tolstykh & Oshchepkova, 2024). At the same time, risks associated with over-reliance remain evident, as dependence on AI-generated explanations can reduce independent processing and limit deeper engagement with texts (Zafar et al., 2025). Earlier work on glossing formats indicates that the design and modality of support continue to shape comprehension outcomes, reinforcing the importance of how assistance is embedded within the reading process (Tadayonifar et al., 2021; Taylor, 2006). These findings converge on a central point: AI reshapes reading from within by altering how attention, effort, and evaluation are distributed, while introducing the possibility that externally generated input may displace independent meaning construction if not carefully regulated.

### 2.4. Timing of Support, Synthesis, and Research Gap

The preceding sections establish a consistent line of argument: reading comprehension in EFL contexts depends on how cognitive effort is allocated across decoding, inferencing, and integration, and this allocation is systematically shaped by external support. Accounts of second-language reading frame comprehension as the construction of a coherent mental representation under conditions of constrained linguistic resources, in which sustained engagement and strategic regulation remain central (Kuperman et al., 2023). Work on instructional support shows that assistance does not simply facilitate performance; it reorganises processing by directing attention, segmenting input, and redistributing effort across comprehension processes (Castro-Alonso et al., 2021; Van Nooijen et al., 2024). Evidence from AI-mediated environments extends this position by demonstrating that support is increasingly embedded in the reading process itself, shaping how readers engage with text in real time rather than after comprehension has occurred (Yuan, 2025; Rad, 2025). These strands converge on a central point: the critical issue is not the presence of support, but the conditions under which it enters and reshapes the process of comprehension.

Within this framework, the timing of support constitutes a theoretically consequential dimension that has not been adequately specified. Immediate access to support during reading allows readers to obtain explanations, glosses, or simplified input at points of difficulty, thereby reducing the processing demands associated with lexical access and integration (Ünal, 2023; Wang et al., 2025). Such access may facilitate task completion, although it can also reduce the need for sustained inferential processing if readers rely on externally generated input. Delayed access introduces a different processing configuration, as readers must engage with the text independently before consulting support, which preserves the requirement to construct meaning through effortful processing (Peng et al., 2024; Smith et al., 2021). The contrast between these configurations concerns when assistance becomes available relative to the reader’s own processing, and therefore whether support functions as a supplement to comprehension or a substitute for it. This distinction has direct implications for the depth of processing and the stability of the resulting mental representation.

Despite its theoretical relevance, the timing of AI-mediated support has not been systematically isolated in empirical research on EFL reading. Existing work has largely examined the effects of support presence, scaffolding type, or learner perceptions, while treating access to assistance as a constant rather than a variable (Fan, 2025; Zafar et al., 2025). In AI-supported environments, assistance is often integrated continuously into the reading process, making it difficult to determine whether observed gains reflect changes in cognitive processing or the availability of immediate support during task performance. As a result, current evidence does not clearly distinguish comprehension arising from independent processing from comprehension facilitated by real-time assistance. This limitation constrains theoretical accounts of AI-mediated reading and restricts the basis for instructional decisions regarding how such tools should be integrated into reading tasks.

The present study addresses this gap by isolating the timing of AI support as the primary independent variable within a controlled experimental design. Rather than examining whether AI support is beneficial in general, the study investigates whether different temporal arrangements of identical AI assistance produce different reading comprehension outcomes. The study holds the form of AI support constant while manipulating only when it becomes available, thereby distinguishing the instructional effects of temporal sequencing from the general effects of AI-assisted learning. This distinction contributes to the growing literature on AI-mediated instruction by demonstrating that the educational value of AI depends not only on the availability of support but also on its temporal integration within the learning process. Although previous literature suggests that support timing may influence cognitive engagement during reading, the present study evaluates this proposition through differences in learning outcomes rather than through direct measurement of the cognitive processes involved. Accordingly, the study addresses the following research question:

- To what extent does the timing of AI support (immediate, delayed, or no support) influence differences in EFL learners’ reading comprehension performance?

To answer this question, the following hypotheses were tested:

**H1.**  *Post-test reading comprehension performance will differ significantly across the three experimental conditions.*

**H2.**  *Learners receiving delayed AI support will obtain higher post-test reading comprehension scores than those receiving immediate AI support.*

**H3.**  *Learners receiving delayed AI support will obtain higher post-test reading comprehension scores than those in the no-AI control group.*

**H4.**  *Learners receiving immediate AI support will obtain higher post-test reading comprehension scores than those in the no-AI control group.*

## 3. Methodology

### 3.1. Research Design

The study employed a pretest–posttest experimental design involving three between-subjects conditions: an Immediate AI Support group, a Delayed AI Support group, and a No-Support control group. Pretest–posttest experimental designs are frequently employed in second-language and instructional-intervention research because they permit direct comparison of treatment effects across instructional conditions while accounting for learners’ initial performance levels (Mackey & Gass, 2022). The design was selected to examine whether variations in the timing of AI-mediated support during reading resulted in statistically significant differences in EFL learners’ post-test reading comprehension performance. The independent variable was the timing of AI support, whereas post-test reading comprehension performance constituted the dependent variable. Participants assigned to the Immediate AI Support condition were permitted to consult the AI tool while reading the assigned text, whereas participants in the Delayed AI Support condition completed the initial reading process independently and accessed the AI tool only after completing the reading task. Participants in the No-Support control condition completed the reading activity without AI assistance. To strengthen internal validity, all groups completed identical reading tasks under comparable instructional conditions, within the same time limits, and using the same assessment procedures. Furthermore, all participants completed the same pre-test before the intervention to establish baseline equivalence in reading comprehension ability across the three experimental groups.

### 3.2. Participants

The participants consisted of 90 undergraduate EFL learners enrolled in the English Language Teaching programme at Fırat University. The sample included both Turkish and international students studying within the same university-level EFL instructional context, where English reading instruction formed part of the participants’ regular academic coursework. The participants ranged in age from 20 to 26 years and included both male and female learners enrolled in the second year of undergraduate study. The participants were selected through convenience sampling from accessible classes within the same institutional setting. Prior to the intervention, all participants completed a reading comprehension pre-test to determine baseline equivalence and to confirm that the learners represented a broadly comparable intermediate level of English reading proficiency. An a priori statistical power analysis was conducted using G*Power 3.1.9.7 to determine the required sample size. The analysis indicated that a minimum of 84 participants was required to detect a medium effect size (*f* = 0.25) at α = 0.05 with statistical power of 0.80. The final sample of 90 participants therefore exceeded the required minimum. Following recruitment, the participants were randomly assigned to one of three experimental conditions: Immediate AI Support, Delayed AI Support, and No-Support control, with 30 learners allocated to each group. Random assignment reduced the likelihood of systematic group differences before the intervention and strengthened the internal validity of the experimental comparison. Participation in the study was voluntary, and only learners who completed both the pre-test and post-test procedures were included in the final analysis.

### 3.3. Instruments and Materials

#### 3.3.1. Reading Comprehension Pre-Test

A reading comprehension pre-test was administered to all participants before the experimental intervention to establish baseline equivalence across the three instructional conditions. The test was based on an academic expository passage titled ‘*Urban Noise and Cognitive Performance*’ and included 10 multiple-choice items designed to assess different levels of reading comprehension (see Supplementary Materials). The passage was selected to be appropriate for intermediate-level university EFL learners in terms of readability, linguistic complexity, and conceptual accessibility. In addition, experienced EFL specialists reviewed the passage to confirm its suitability for the target population and its overall comprehension demands. The instrument comprised four literal comprehension items targeting explicitly stated information, three inferential comprehension items requiring learners to derive implicit meaning and integrate information across the text, and three global comprehension items assessing understanding of the passage as a whole, including its central ideas and overall message. Each correct response was worth one point, for a maximum score of 10, with higher scores indicating stronger reading comprehension. The resulting scores were used to assess baseline comparability among the three experimental groups prior to the instructional treatment.

#### 3.3.2. Reading Materials Used During the Intervention

The reading materials used during the intervention consisted of academic expository passages developed for intermediate-level EFL learners within a university instructional context. The passages were selected and adapted to maintain broadly similar levels of length, readability, lexical difficulty, and syntactic complexity across the intervention period. To strengthen comparability, the passages were also reviewed by experienced EFL specialists, who evaluated their overall linguistic complexity, readability, and comprehension demands to ensure that the materials were appropriate and comparable for intermediate-level university EFL learners. Particular attention was given to controlling textual variation so that differences in reading comprehension performance could be interpreted more confidently as reflecting the effects of the instructional treatment rather than inconsistencies in passage difficulty. The texts addressed general academic topics that were accessible to university-level learners and did not require specialised disciplinary knowledge, thereby reducing the potential influence of background knowledge on comprehension. In addition, the passages were designed to support engagement with multiple levels of comprehension, including literal understanding, inferential comprehension, and global interpretation. All intervention materials were administered under identical instructional conditions and within the same time limits across the three experimental groups to maintain procedural consistency throughout the study.

#### 3.3.3. Reading Comprehension Post-Test

The post-test was administered immediately after the intervention to examine differences in reading comprehension performance across the three instructional conditions. The test was constructed around an academic expository passage addressing plastic pollution in marine ecosystems (see Supplementary Materials). The topic was selected because it was appropriate for university-level EFL learners while remaining conceptually independent of the instructional intervention. Care was taken to select a passage that was broadly similar to the pre-test text in length, linguistic complexity, and overall readability in order to minimise variation in textual difficulty. The passage was also reviewed by the same EFL specialists to ensure that it was comparable to the pre-test passage in terms of linguistic complexity, readability, and overall comprehension demands while addressing a different topic to minimise practice effects. The post-test included ten multiple-choice comprehension items distributed across three dimensions of reading comprehension. Four items assessed literal comprehension through the identification of explicitly stated information, three items measured inferential comprehension by requiring learners to interpret implied meaning and establish relationships among ideas presented in the text, and three items assessed global comprehension through recognition of the main argument and broader interpretation of the passage. The inclusion of multiple comprehension levels allowed the assessment to examine reading performance beyond simple information recall. Although the post-test measured the same underlying comprehension constructs as the pre-test, a different reading passage was used to minimise familiarity effects associated with repeated exposure to the same text. Each correct response was awarded one point, producing a maximum possible score of 10, with higher scores indicating stronger reading comprehension. The post-test was administered under identical classroom conditions, using the same instructions, time limits, and scoring procedures across all experimental groups.

#### 3.3.4. AI Support System

The experimental treatment was implemented using ChatGPT (GPT-5.2; OpenAI), with participants accessing the publicly available web-based ChatGPT interface. During the data collection period, GPT-5.2 was the default ChatGPT model available on the platform. The system provided comprehension assistance during the reading task by allowing participants to type questions and receive immediately generated responses related to the reading passage. It was used primarily for vocabulary clarification, explanation of sentence meaning, summarisation of textual information, and clarification of comprehension difficulties encountered during reading. To maintain consistency across the intervention, all participants in the AI-supported conditions used the same AI platform, followed identical classroom procedures, and completed the reading activities within the same time limits.

The timing of AI access differed across the two experimental conditions. Participants assigned to the Immediate AI Support condition were permitted to consult the AI tool while reading the passage and while answering comprehension questions. In contrast, participants in the Delayed AI Support condition completed the initial reading independently, without AI assistance, and were allowed to access the AI tool only after finishing the reading task. This distinction was implemented to examine whether immediate access to AI-generated explanations altered learners’ engagement with text processing in ways different from delayed access after independent reading. Participants in the No-Support control condition completed the same reading tasks without AI assistance.

To reduce procedural variation among participants, the instructional procedures governing AI use were standardised prior to the intervention. Participants in the AI-supported conditions were informed that the system could be used only for comprehension support related to the assigned reading passage. Specifically, AI use was limited to vocabulary clarification, explanation of sentence meaning, summarisation of textual information, and clarification of comprehension difficulties arising from the assigned reading passage. The same instructions regarding AI use, reading time, and task completion procedures were applied consistently across the two AI-supported groups. In addition, all intervention sessions were conducted under one of the researchers’ supervision to monitor adherence to the assigned condition and to minimise cross-condition contamination during the experimental procedure.

Participants in the AI-supported conditions received identical instructions on using the system before the intervention began. They were informed that ChatGPT was intended solely to support comprehension of the assigned reading passages and was not permitted to provide direct answers to the comprehension questions. Participants were explicitly instructed not to request answers to the assessment items or reproduce AI-generated responses in their test answers. To preserve relatively natural interaction with the reading materials, participants were permitted to formulate their own comprehension-related queries rather than follow a fixed set of prompts. Typical queries included requesting the meaning of unfamiliar words, asking for clarification of sentence or paragraph meaning, requesting summaries of sections of the reading passage, or asking for explanations of relationships between ideas presented in the text. Participants were also instructed to begin each reading session by initiating a new ChatGPT conversation. Consequently, conversation history from previous sessions was not retained, ensuring that each reading activity was completed independently under equivalent initial conditions. This approach was adopted to reflect authentic AI-assisted reading practices while maintaining consistency in the instructional purpose of AI use across participants through standardised guidance and identical task requirements. All sessions were conducted under classroom supervision, and participants were reminded throughout the intervention to adhere to the assigned procedures and use the system only for reading-related support. Although participants’ prompt histories, interaction frequency, interaction duration, and AI-generated responses were not recorded as part of the experimental protocol, the use of identical instructions, the same AI platform, equivalent reading materials, fixed time limits, and continuous classroom supervision were intended to maximise procedural consistency across the AI-supported conditions while preserving the ecological validity of authentic AI-supported learning. All groups completed the intervention within the same instructional period and under identical time limits.

### 3.4. Validation Procedures

Prior to the main experiment, the reading comprehension instrument was piloted with a separate sample of 157 EFL learners comparable to the target study population in order to examine item functioning, internal consistency, and measurement precision across the different dimensions of reading comprehension. The validation analysis was conducted in R version 4.3.1 using the openxlsx package for data importation and the mirt package for item response modelling. The validation incorporated procedures derived from both Classical Test Theory (CTT) and Item Response Theory (IRT) to provide complementary evidence regarding the reliability, item functioning, and measurement precision of the instrument before its administration in the main experiment. The CTT analysis examined item difficulty, point-biserial correlations, and internal reliability, whereas the IRT analysis examined item difficulty, item discrimination, infit and outfit statistics, and measurement precision across the latent reading comprehension continuum. Additional graphical analyses were conducted using item characteristic curves, item information curves, the test information function, and the person-item map to examine item functioning across different levels of reading comprehension ability. After completing the validation procedures, the final version of the reading comprehension instrument was retained for administration in the main experimental study. The combination of CTT and IRT analyses allowed the instrument to be evaluated from complementary psychometric perspectives, providing evidence that the retained items functioned appropriately for assessing reading comprehension in the target population. Section 4 presents detailed validation findings.

### 3.5. Experimental Procedures

The experimental intervention was implemented over a ten-week period during the second term of the 2025–2026 academic year within the participants’ university EFL programme. Participants attended two reading sessions per week, with each session lasting approximately 50 min. The intervention, therefore, consisted of 20 instructional sessions conducted over the treatment period. Prior to the intervention, all participants completed the reading comprehension pre-test under identical classroom conditions and within the same time limits in order to establish baseline equivalence across the three experimental groups. Following the pre-test administration, participants were randomly assigned to one of three instructional conditions: Immediate AI Support, Delayed AI Support, or No-Support control. The same instructor-researcher conducted and supervised all intervention sessions across the three groups to reduce procedural variation in instructional delivery.

Before the intervention began, participants assigned to the AI-supported conditions received a brief orientation on using the AI system and the procedures governing the reading activities. The intervention consisted of individual reading comprehension tasks based on academic expository passages administered throughout the treatment period. Different reading passages of comparable length, readability level, lexical difficulty, and syntactic complexity were used across the intervention sessions in order to maintain consistency in textual difficulty throughout the study. The AI-supported conditions used the latest version of ChatGPT accessed through a web-based interface. Participants used the system through their personal devices under classroom supervision. Participants assigned to the Immediate AI Support condition were permitted to access the AI system throughout the reading process and while responding to the comprehension questions. They were allowed to consult the system for vocabulary clarification, explanations of sentence meaning, summarisation of textual information, and comprehension-related support for the assigned reading passage. In contrast, participants assigned to the Delayed AI Support condition first completed the reading tasks independently, without AI assistance, and were granted access to the AI system only after completing the initial reading tasks. This procedure was implemented to examine whether postponing AI-generated assistance promoted greater independent cognitive engagement before technological support became available. Participants in the No-Support control condition completed the same reading activities and comprehension tasks without AI assistance at any stage of the intervention.

To maintain consistency across experimental conditions, all participants completed reading tasks of comparable difficulty, received identical instructions, and were allocated the same reading and response time throughout the intervention period. To reduce procedural variation in AI use across participants, learners in the AI-supported conditions received the same instructions regarding permissible AI use, and AI consultation was restricted to comprehension-related support for the assigned reading passages, including vocabulary clarification, sentence-level explanation, summarisation, and clarification of textual meaning. The prompts used in the AI-supported conditions were intentionally not fully standardised because the objective of the study was to isolate the effects of AI support timing while allowing participants to formulate comprehension-related queries that reflected their individual reading needs. Nevertheless, all participants followed identical instructional guidelines, completed the same reading tasks under the same classroom conditions, used the same AI platform, and were allocated identical reading and response times. Participants were instructed to use the AI system only for comprehension-related support for the assigned texts. They were further instructed not to request direct answers to the comprehension questions or reproduce AI-generated responses directly in their answers. All intervention sessions were conducted under the direct supervision of the instructor-researcher, who supervised the implementation of the experimental procedures, reminded participants throughout the intervention to follow the established AI-use guidelines, and addressed procedural questions when necessary. However, participants’ prompt histories, interaction frequency, interaction duration, and AI-generated responses were not recorded as part of the experimental protocol. Consequently, although procedural consistency was promoted through standardised instructions, identical instructional procedures, and classroom supervision, individual differences in AI usage behaviour could not be quantified and are acknowledged as a limitation when interpreting the findings. Access to unrelated online materials and unrestricted internet browsing beyond the assigned AI platform was not permitted during the intervention. Immediately after the treatment period, all participants completed the reading comprehension post-test under the same classroom procedures, instructions, and time constraints. After data collection was completed, the responses were coded and prepared for statistical analysis.

### 3.6. Ethical Considerations

Ethical approval for the study was obtained from the Social and Human Sciences Research Ethics Committee at Fırat University prior to the commencement of data collection (Session No. 2026/6; Decision No. 2). Participation in the study was voluntary, and all participants were informed about the purpose of the research, the nature of the experimental procedures, and their right to withdraw from the study at any stage without academic penalty. Written informed consent was obtained from all participants before the intervention began. To protect participant privacy, all responses were coded anonymously, and no personally identifiable information was included in the dataset or subsequent analyses. The study was conducted in accordance with established ethical principles governing research involving human participants.

### 3.7. Data Analysis

The data analysis was conducted in two stages using R version 4.3.1 and IBM SPSS Statistics version 29. The first stage involved psychometric validation of the reading comprehension instrument using procedures based on both CTT and IRT. The *openxlsx* package was used for data importation, whereas the *mirt* package was employed for item response modelling. The validation analysis examined item difficulty, point-biserial correlations, Cronbach’s alpha, item discrimination, infit and outfit statistics, item characteristic curves, item information curves, the test information function, and the person-item map to evaluate item functioning and measurement precision across the latent reading comprehension continuum. Following completion of the validation procedures, the main statistical analyses were conducted in SPSS. Preliminary analyses were conducted to examine the assumptions underlying a one-way analysis of variance (ANOVA). Residual normality was assessed using the Shapiro–Wilk test and visual inspection of diagnostic plots; homogeneity of variance was examined using Levene’s test; linearity between the covariate and outcome was assessed; and the homogeneity of regression slopes was evaluated through the interaction between instructional condition and pre-test performance. After these assumptions were found to be adequately satisfied, ANCOVA was conducted to examine differences in post-test reading comprehension across the three instructional conditions while controlling for pre-test reading comprehension. Estimated marginal means were calculated to describe adjusted post-test performance across the conditions, and Bonferroni-adjusted pairwise comparisons were used to identify specific differences between groups. Partial eta squared (η^2^p) was reported as the effect-size measure for the ANCOVA, and 95% confidence intervals were reported for the adjusted means and pairwise differences. Descriptive statistics, including means and standard deviations, were also calculated for pre-test and post-test reading comprehension scores. Statistical significance was evaluated at *p* < .05 throughout the analysis.

## 4. Results

### 4.1. Validation of the Reading Comprehension Test

Prior to the main experiment, a psychometric validation analysis was conducted on the pilot sample responses in order to examine the functioning of the reading comprehension instrument. The analysis combined procedures derived from CTT and IRT using R version 4.3.1. The *openxlsx* package was used to import the dataset, whereas the mirt package was used for item response modelling. The CTT analysis first examined the performance of the ten comprehension items across the literal, inferential, and global comprehension dimensions (Table 1). Item difficulty values ranged from 0.291 to 0.826, indicating variation in comprehension demand across the instrument rather than concentration around a narrow difficulty range. Literal comprehension items generally produced higher facility values, particularly Lit1 and Lit2, whereas the global comprehension items, especially Glo2 and Glo3, required comparatively greater processing demand. This distribution corresponds to the conceptual organisation of the instrument because literal comprehension relies primarily on retrieving explicitly stated textual information, whereas global comprehension requires broader integration of ideas across the passage.

Point-biserial correlations ranged from 0.376 to 0.501, with all items exceeding the commonly accepted minimum value of 0.30. The pattern indicates that all items contributed positively to differentiating between learners with higher and lower levels of reading comprehension. Internal consistency analysis produced a Cronbach’s alpha coefficient of 0.762, which is acceptable for a classroom-based reading comprehension measure. The drop-alpha coefficients ranged from 0.731 to 0.754, and none exceeded the overall reliability coefficient, indicating no problematic item functioning or need for item removal. The CTT findings consequently support retaining all ten comprehension items for the subsequent stages of the analysis.

The analysis subsequently proceeded to IRT modelling in order to examine item functioning across the latent reading comprehension continuum. A two-parameter logistic model was estimated because it permits item difficulty and item discrimination to vary across items, whereas the one-parameter Rasch model was also estimated for graphical comparison. The IRT difficulty estimates ranged from −1.625 to 1.407, indicating that the instrument covered a relatively broad range of learner ability levels (Table 2). The easiest items were Lit2 and Lit1, both belonging to the literal comprehension dimension, whereas Glo3, Glo2, and Glo1 produced the highest difficulty estimates. The distribution again reflects the instrument’s conceptual structure: literal comprehension relies primarily on retrieving explicitly stated information, whereas global comprehension requires broader integration and interpretation across the text. The item fit statistics similarly remained within acceptable ranges across the instrument. Outfit mean-square values ranged from 0.702 to 0.951, whereas infit mean-square values ranged from 0.786 to 0.965, indicating stable item functioning across the latent ability continuum.

The discrimination coefficients ranged from 0.742 to 1.654 and remained positive across all items. Most items produced moderate-to-strong discrimination indices, indicating acceptable differentiation between learners with lower and higher levels of reading comprehension. Although Inf2 produced the lowest discrimination coefficient, the value remained acceptable for a short classroom-based comprehension instrument. The combined pattern of item difficulty, item discrimination, and fit statistics therefore supports the instrument’s psychometric adequacy across the different dimensions of reading comprehension.

**Figure 1:**
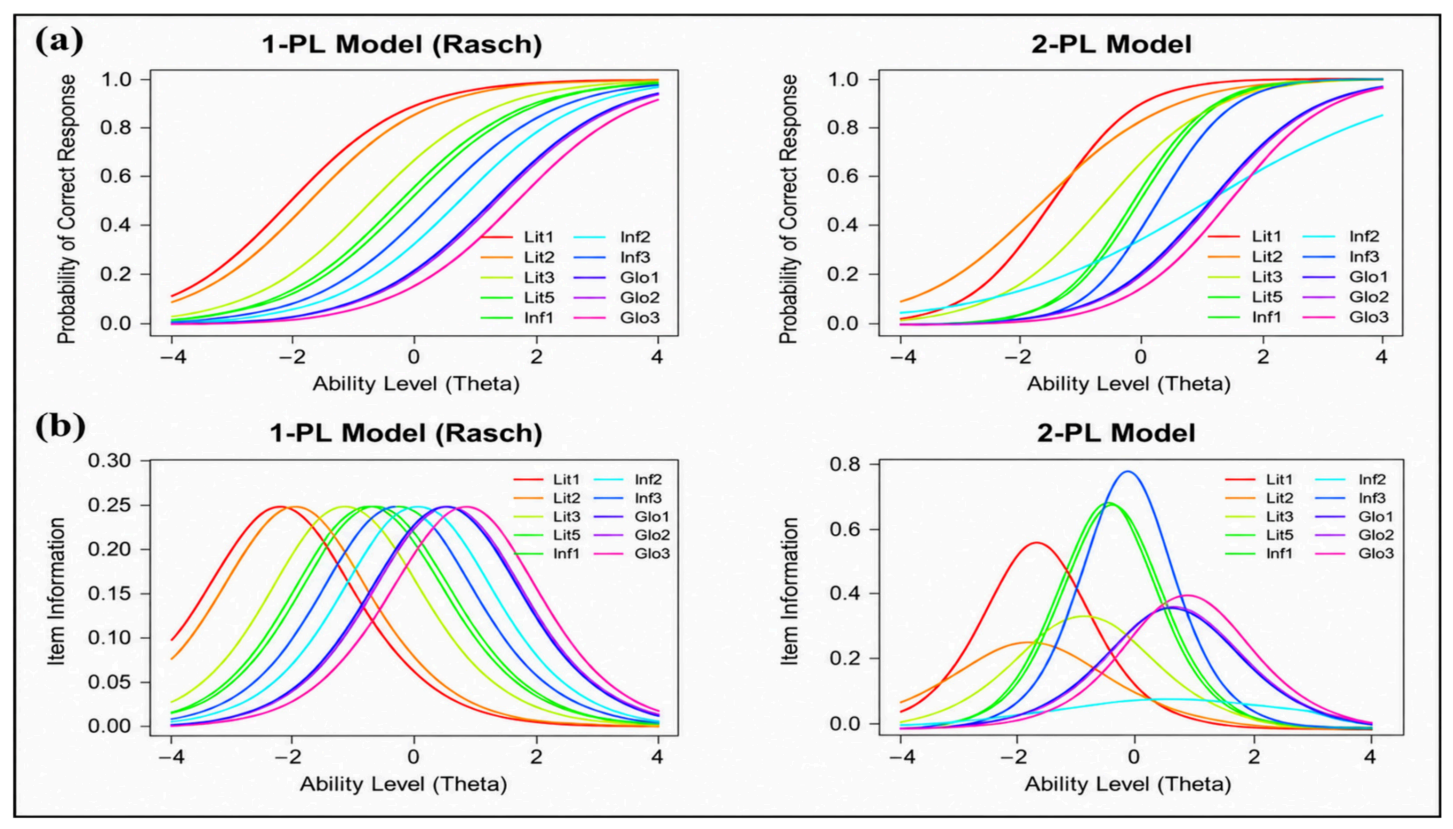
ICC (**a**) and IIC plots (**b**) across 1-PL and 2-PL.

The graphical IRT analyses provide additional insight into how the reading comprehension items functioned across different levels of learner ability. The item characteristic curves (ICC) produced the expected S-shaped response patterns under both the Rasch and 2-PL models (Figure 1a). Under the Rasch model, the curves appeared relatively parallel because item discrimination was constrained to remain equal across items. In contrast, the 2-PL model produced clearer variation in curve steepness because discrimination parameters were estimated freely for each item. Items such as Inf3, Inf1, and Lit5 generated comparatively steeper slopes, indicating sharper differentiation between learners located around nearby ability levels, whereas Inf2 produced a flatter response pattern corresponding to its comparatively lower discrimination value. The graphical distribution of the curves also reflects the instrument’s internal structure, with literal comprehension items positioned in the lower regions of the ability continuum and global comprehension items concentrated at comparatively higher levels of reading comprehension ability.

The item information curves (IIC) further illustrate how measurement precision varied across the instrument (Figure 1b). Under the Rasch model, the information curves appeared more evenly distributed because discrimination was held constant across items. The 2-PL model produced greater variation in information peaks, particularly for items with stronger discrimination values. Several items generated concentrated peaks in the low-to-middle and middle regions of the ability continuum, indicating greater measurement precision in those ranges. In contrast, some items contributed broader, less concentrated information across wider ranges of learner ability. The graphical pattern suggests that the instrument functioned most effectively for learners with low-to-moderate and moderate levels of reading comprehension ability rather than at the extreme upper end of the continuum.

**Figure 2:**
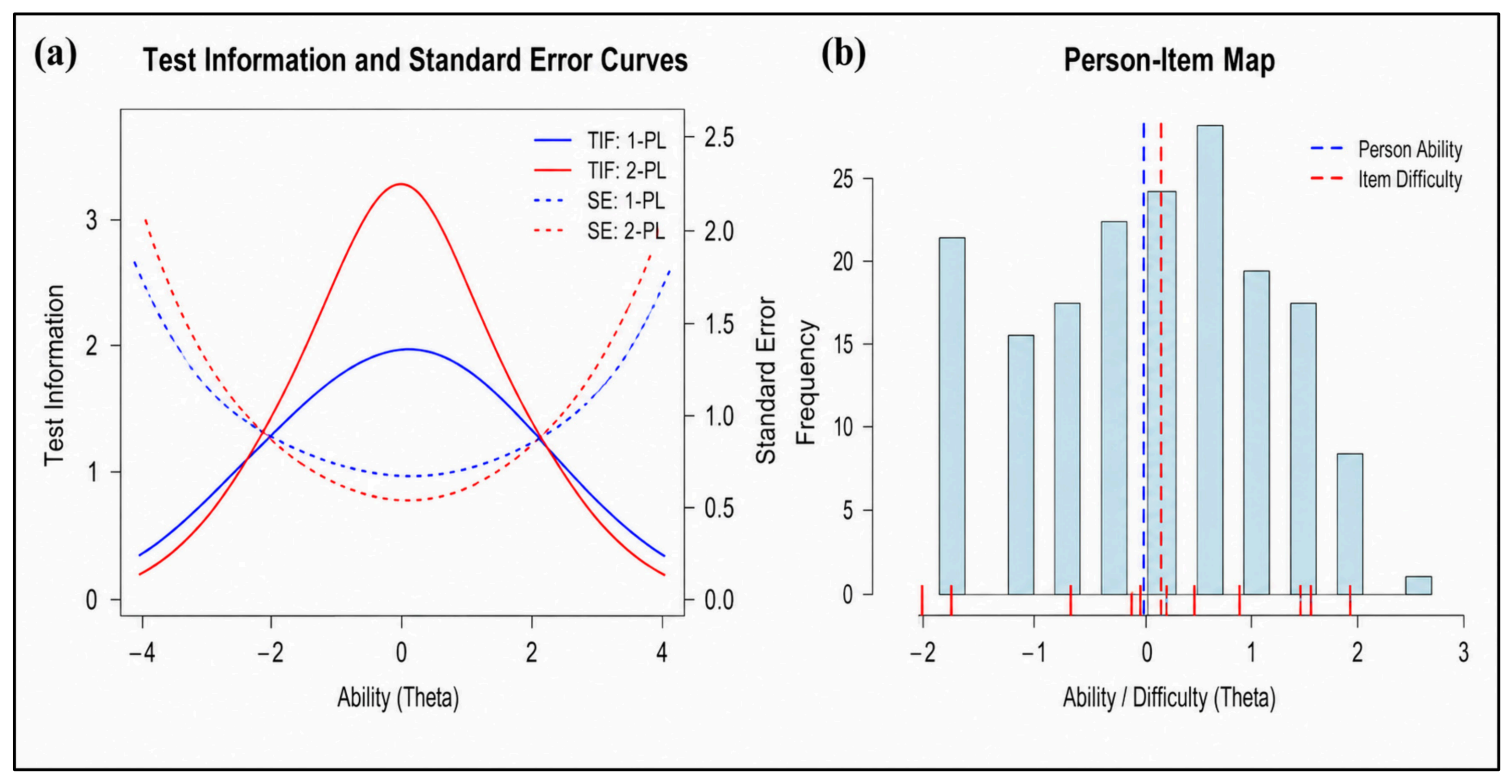
Plot of TIF (**a**) and person item map (**b**).

The test information curves indicate that the instrument produced its highest measurement precision within the low-to-middle and middle regions of the ability continuum (Figure 2a). Standard error values decreased in regions where test information increased and became larger toward the extreme ends of the scale. The instrument therefore functioned more effectively for learners with low-to-moderate and moderate reading comprehension ability than for highly proficient readers. This distribution is appropriate given the proficiency profile of the present sample, which consisted primarily of intermediate-level university EFL learners.

The person-item map further illustrates the relationship between learner ability estimates and item difficulty estimates along the same theta continuum (Figure 2b). Item difficulty was distributed reasonably well across the scale, with the literal comprehension items clustered at lower difficulty levels and the global comprehension items positioned at comparatively higher regions of the continuum. Inferential comprehension items occupied intermediate regions of the scale. The distribution suggests that the instrument was capable of differentiating learners across multiple levels of reading comprehension ability without excessive concentration around a narrow difficulty range. The relatively limited number of highly difficult items, nevertheless, indicates comparatively lower measurement sensitivity at the upper end of the continuum.

### 4.2. Preliminary Analyses

Prior to hypothesis testing, the assumptions underlying ANCOVA were examined. The residuals were approximately normally distributed, as indicated by the non-significant Shapiro–Wilk test, W = 0.989, *p* = .673. Levene’s test was also non-significant, F(2, 87) = 0.091, *p* = .914, supporting homogeneity of variance. The relationship between pre-test and post-test reading comprehension was adequately linear, with no significant quadratic deviation, F(1, 85) = 0.741, *p* = .392. Finally, the interaction between instructional condition and pre-test performance was non-significant, F(2, 84) = 0.440, *p* = .646, supporting the homogeneity of regression slopes assumption. Overall, the preliminary analyses indicated that the assumptions for ANCOVA were adequately met.

**Figure 3:**
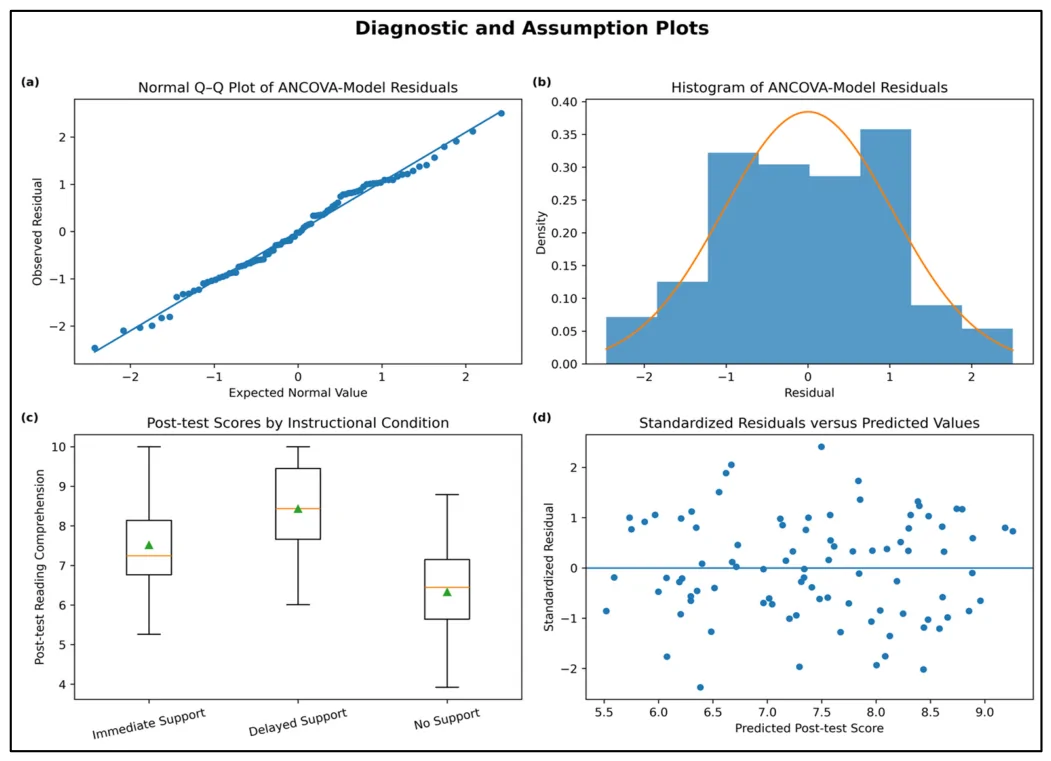
Diagnostic and assumption plots for the ANCOVA model: (**a**) normal Q–Q plot of ANCOVA-model residuals; (**b**) histogram of ANCOVA-model residuals with an overlaid normal density curve; (**c**) boxplots of post-test reading comprehension scores by instructional condition; (**d**) standardized residuals plotted against predicted post-test scores.

As shown in Figure 3, visual inspection of the diagnostic plots provided further support for the ANCOVA assumptions. The residuals approximated a normal distribution, with no serious outliers or systematic pattern across predicted values. These graphical results were consistent with the preliminary tests reported in Table 3 and supported proceeding with the ANCOVA.

### 4.3. Hypotheses Testing

An ANCOVA was conducted to examine differences in post-test reading comprehension across the three instructional conditions while controlling for pre-test performance. Table 4 presents the descriptive statistics for the pre-test and post-test scores. Pre-test means were similar across the Immediate Support (M = 7.19, SD = 1.01), Delayed Support (M = 7.21, SD = 1.05), and No Support (M = 7.08, SD = 1.04) conditions. At post-test, the Delayed Support condition had the highest unadjusted mean (M = 8.43, SD = 1.14), followed by the Immediate Support condition (M = 7.51, SD = 1.16) and the No Support condition (M = 6.32, SD = 1.11).

As shown in Table 5, the ANCOVA revealed a significant effect of instructional condition on post-test reading comprehension after controlling for pre-test performance, F(2, 86) = 28.36, *p* < .001, partial η^2^ = 0.397. Pre-test performance was also significantly related to post-test scores, F(1, 86) = 14.67, *p* < .001, partial η^2^ = 0.146. Overall, the model accounted for 46.5% of the variance in post-test reading comprehension (R^2^ = 0.465, adjusted R^2^ = 0.446).

The estimated marginal means presented in Table 6 show that, after controlling for pre-test performance, the Delayed Support condition had the highest adjusted post-test mean (M = 8.41, SE = 0.19, 95% CI [8.03, 8.79]), followed by the Immediate Support condition (M = 7.50, SE = 0.19, 95% CI [7.12, 7.88]) and the No Support condition (M = 6.36, SE = 0.19, 95% CI [5.97, 6.74]).

The Bonferroni-adjusted pairwise comparisons (Table 7) showed that the Delayed Support condition significantly outperformed the Immediate Support condition (MD = 0.91, SE = 0.27, *p* = .004, 95% CI [0.24, 1.57]) and the No Support condition (MD = 2.05, SE = 0.27, *p* < .001, 95% CI [1.39, 2.72]). The Immediate Support condition also significantly outperformed the No Support condition (MD = 1.14, SE = 0.27, *p* < .001, 95% CI [0.48, 1.81]). These comparisons confirm that adjusted post-test performance was highest under Delayed Support, followed by Immediate Support and No Support.

Overall, the results supported all four hypotheses. Instructional condition had a significant effect on post-test reading comprehension after controlling for pre-test performance (H1). The pairwise comparisons further showed that the Delayed Support condition significantly outperformed both the Immediate Support condition (H2) and the No Support condition (H3), while the Immediate Support condition significantly outperformed the No Support condition (H4). Thus, the pattern of adjusted post-test performance was consistent across the analyses, with Delayed Support yielding the highest reading comprehension scores, followed by Immediate Support and No Support.

## 5. Discussion

### 5.1. Differences Across the Three Experimental Conditions

The findings support H1, indicating statistically significant differences in post-test reading comprehension performance across the three instructional conditions. Learners who received delayed AI support achieved the highest post-test scores, whereas the no-support group produced the weakest performance. The immediate support group occupied an intermediate position between the two conditions. This pattern suggests that the instructional effects of AI support were related to when explanatory assistance became available during the reading process. Learners in the delayed support condition were required to interpret unfamiliar vocabulary, establish relationships across ideas, and address comprehension difficulty independently before consulting the AI system. Alternative explanations are also plausible. For example, learners in the delayed support condition may have developed greater awareness of their own comprehension difficulties during the initial independent reading, allowing them to use AI-generated assistance more selectively when it became available. Likewise, learners in the immediate support condition may have experienced greater attentional switching as they alternated between reading the passage and consulting the AI system. Because these processes were not directly examined, the present study cannot determine which explanation best accounts for the observed differences. The AI system, consequently, may have functioned primarily as supplementary support following an initial attempt at understanding the text rather than as an immediate interpretive resource during reading itself. Learners in the immediate support condition, in contrast, could access explanatory assistance while their comprehension difficulties were still unfolding. Although this form of support may have reduced disruption during reading, it may also have reduced opportunities for learners to persist independently in the face of lexical or conceptual uncertainty before seeking external clarification. Previous research on second-language reading has consistently linked stronger comprehension performance to inferencing, monitoring, and the integration of textual information during reading (Smith et al., 2021).

The findings similarly align with research examining instructional support and cognitive processing during reading tasks. Cognitive load research has argued that instructional assistance facilitates comprehension most effectively when support redirects attention toward relevant textual information without replacing learners’ own engagement with the reading task (Castro-Alonso et al., 2021; Peng et al., 2024). The comparatively stronger performance observed in the delayed support condition is consistent with this theoretical perspective, although the present findings cannot determine whether differences in cognitive engagement or processing were responsible for the observed performance advantage because these constructs were not directly assessed. At the same time, both AI-supported groups outperformed the no-support condition, indicating that AI-generated explanations and clarifications were associated with improved management of comprehension difficulty during second-language reading. Earlier research has likewise linked AI-supported reading environments with stronger strategic engagement and reading performance among EFL learners (Bahari et al., 2023; Fan, 2025; Pan et al., 2025). Accordingly, the present study contributes by demonstrating that the timing of AI support is associated with different reading comprehension outcomes under controlled experimental conditions. However, the cognitive mechanisms responsible for these differences remain uncertain, and future research should directly examine competing explanations, including independent cognitive engagement, problem awareness, attentional switching, and other process-level factors that may influence learners’ use of AI during reading.

### 5.2. Delayed AI Support and Reading Comprehension Performance

Support for H2 and H3 was reflected in the significantly higher post-test reading comprehension scores achieved by learners in the delayed support condition than by those in the immediate support and no-support groups. The pattern suggests that delayed AI-assisted reading created conditions in which learners were required to engage with textual difficulty independently before external clarification became available. Learners in the delayed support condition completed the initial stages of comprehension without AI-generated assistance, which may have provided greater opportunities for learners to rely on their own linguistic knowledge and interpretive resources during reading. However, because the present study did not directly assess cognitive effort, cognitive load, or metacognitive processing, this interpretation remains theoretically informed rather than empirically verified. Alternative explanations should also be considered. For example, learners in the delayed support condition may have developed greater awareness of specific comprehension difficulties during the initial independent reading, enabling them to consult the AI system more purposefully once support became available. Similarly, the superior performance associated with delayed support may reflect more effective timing of AI consultation rather than differences in cognitive engagement alone. Because these processes were not directly examined, the present study cannot determine which explanation best accounts for the observed differences. Under these conditions, AI-generated clarification may have functioned primarily as supplementary support following an initial attempt at understanding the text rather than as continuous interpretive guidance throughout the reading process. Learners in the immediate support condition, in contrast, could access explanatory assistance as comprehension difficulties were unfolding, whereas learners in the no-support condition remained dependent on their existing linguistic knowledge and comprehension strategies throughout the task. The comparatively weaker performance observed across these two conditions may therefore reflect different forms of comprehension constraint. Immediate support may have reduced the extent to which learners attempted to resolve textual ambiguity independently before consulting external clarification, whereas the absence of support in the control condition may have increased the likelihood that comprehension difficulties remained unresolved when learners encountered unfamiliar vocabulary, syntactic complexity, or conceptually demanding sections of the text. For example, learners receiving immediate AI support may have experienced greater attentional switching as they alternated between reading the text and consulting the AI system, whereas learners in the no-support condition may have remained unable to resolve comprehension difficulties when encountering unfamiliar vocabulary, syntactic complexity, or conceptually demanding sections of the text. These interpretations remain plausible but cannot be confirmed using the present data.

The findings correspond with previous research associating stronger second-language reading comprehension with inferential engagement, comprehension monitoring, and active integration across textual information (Samiei & Ebadi, 2021). Research grounded in cognitive load theory has similarly argued that instructional support facilitates comprehension most effectively when assistance redirects learners toward relevant textual information without replacing processes ordinarily performed during independent comprehension (Castro-Alonso et al., 2021; Skulmowski & Xu, 2022). Earlier studies examining AI-supported language learning have likewise associated AI-mediated explanation and feedback with stronger strategic engagement and improved management of linguistic complexity during reading activities (Bahari et al., 2023; Fan, 2025; Pan et al., 2025). The present findings extend this line of research by suggesting that the instructional value of AI support may depend substantially on the stage at which explanatory assistance becomes available during reading. Although the superior performance observed under delayed support is consistent with theoretical accounts that emphasise independent processing before external assistance, the present experiment evaluated learning outcomes rather than the cognitive mechanisms underlying those outcomes. Accordingly, the findings should be interpreted as identifying an association between the timing of AI support and reading comprehension performance rather than demonstrating the specific cognitive processes responsible for this relationship. Future research incorporating direct measures of cognitive processing and AI interaction behaviour is needed to determine why delayed support produces superior reading comprehension performance.

### 5.3. Immediate AI Support Versus No AI Support

The findings for H4 showed that learners who received immediate AI support during reading achieved significantly higher post-test reading comprehension scores than those in the no-support condition. The difference between the two groups suggests that real-time explanatory assistance was associated with fewer disruptions to comprehension while learners progressed through the text. Learners in the immediate support condition could consult AI-generated clarification when encountering unfamiliar vocabulary, unclear references, or conceptually demanding sections, which may have helped them maintain greater continuity of comprehension during reading. In second-language reading, unresolved lexical or conceptual difficulties can disrupt overall comprehension because difficulties encountered at one stage of reading may influence the interpretation of subsequent textual information. Immediate access to clarification may therefore have assisted learners in addressing comprehension difficulties before they accumulated in later stages of the reading task. However, alternative explanations are also possible. For example, the availability of immediate AI support may have reduced uncertainty by providing timely clarification, whereas the observed performance advantage may also reflect differences in how learners decided when and why to consult the AI system. Because these processes were not directly examined, the present study cannot determine which explanation best accounts for the observed differences between the immediate-support and no-support conditions. Learners in the no-support condition, in contrast, remained dependent on their existing linguistic knowledge and inferential resources throughout the task. When comprehension difficulties arose, learners had no supplementary source of clarification to help them revise incomplete or inaccurate interpretations. The comparatively weaker performance observed in the no-support condition is consistent with the possibility that unresolved comprehension difficulties accumulated during reading; however, this interpretation was not directly examined through measures of cognitive processing or comprehension monitoring.

The findings correspond with previous research linking instructional scaffolding with stronger reading comprehension performance in second-language contexts characterised by linguistic complexity and restricted lexical familiarity (Grabe & Stoller, 2020). Earlier work on AI-assisted language learning has likewise linked AI-generated explanations and clarifications to improved comprehension monitoring and reduced interruptions during reading activities (Bahari et al., 2023; Zhang & Zou, 2024). The present findings suggest that immediate AI support was associated primarily with maintaining continuity of comprehension during reading rather than with the more sustained independent engagement with the reading task observed in the delayed support condition. This interpretation is consistent with existing theoretical perspectives but represents only one plausible explanation for the observed findings rather than a demonstrated cognitive mechanism. Because the present study did not directly examine the processes underlying learners’ interactions with AI, alternative explanations, including differences in strategic AI use, problem awareness, or attentional allocation, cannot be ruled out. Accordingly, the findings should be interpreted as demonstrating that immediate AI support was associated with higher reading comprehension performance than unsupported reading under the conditions of this experiment, while the mechanisms underlying this relationship remain to be established through future research.

## 6. Theoretical and Practical Implications

The findings contribute to the growing literature on AI-assisted language learning by demonstrating that the instructional effects of AI support depend not only on its availability but also on when that support is introduced during the reading process. By experimentally isolating the timing of AI support while holding the form of AI assistance constant, the study identifies temporal sequencing as an instructional variable associated with differences in reading comprehension performance. Although the present study was not designed to identify the cognitive mechanisms underlying these differences, the findings provide an empirical basis for future research examining how the timing of AI support influences cognitive processing during reading. Learners who received delayed AI assistance achieved stronger comprehension performance than those who accessed support during reading, indicating that delayed access to identical AI support was associated with superior reading comprehension outcomes. One possible interpretation is that postponing explanatory assistance gave learners more opportunities to engage independently with the text before consulting external support; however, this explanation remains theoretically informed because cognitive effort, cognitive load, and metacognitive processing were not directly measured. Immediate support, although beneficial relative to unsupported reading, was associated with a different pattern of learning outcomes because explanatory assistance was available while comprehension difficulties were still unfolding. Accordingly, the findings suggest that the timing of AI support represents an important instructional consideration in AI-assisted reading environments and warrants explicit attention in future theoretical and empirical work.

The findings can also be considered in relation to classical and contemporary accounts of intelligence. Classical psychometric models, particularly the Cattell–Horn–Carroll framework, recognise acquired knowledge and language-related abilities as important components of intellectual performance and therefore provide a relevant basis for considering reading comprehension as a cognitively demanding activity. The present findings point to an additional issue: performance may depend partly on how external cognitive support is introduced while learners are engaged in such a task. Delayed AI support required learners to engage with the text before assistance became available, whereas immediate support allowed external assistance during the initial processing of textual information. This distinction is relevant to contemporary views of intelligence that give greater attention to how cognitive resources are used and regulated in response to task demands. In this respect, AI may function as an external resource whose value depends partly on when it is made available in relation to learners’ own processing. The findings do not indicate that the timing of AI support changes intelligence itself, nor was intelligence directly assessed in the present study. Rather, they raise questions about how individual cognitive abilities may relate to the effective use of AI assistance. Future research could examine whether verbal comprehension, working memory, metacognitive regulation, or other individual differences moderate the effects of immediate and delayed AI support.

The findings also carry implications for the organisation of AI-supported reading activities in EFL instruction. The comparatively weaker performance observed in the no-support condition suggests that the absence of explanatory mediation may leave learners vulnerable to unresolved lexical and conceptual difficulty during reading tasks. At the same time, the superiority of delayed support over immediate support suggests that unrestricted real-time access to AI-generated explanations may reduce opportunities for learners to engage independently with challenging textual material before seeking external clarification. Consequently, the findings suggest that AI-assisted reading tasks may be more effective when learners first attempt to process textual meaning independently before seeking supplementary clarification. This recommendation is based on the observed differences in learning outcomes across the experimental conditions rather than on direct evidence regarding the cognitive mechanisms responsible for those differences. This pattern appears particularly relevant in reading contexts that require inferential processing and integration of multiple ideas, rather than surface-level retrieval of textual information.

## 7. Limitations and Directions for Further Research

Several limitations should be acknowledged. First, although the theoretical interpretation of the findings was informed by perspectives on cognitive effort, instructional scaffolding, and independent meaning construction, the present study was designed to evaluate differences in reading comprehension performance rather than to identify the cognitive mechanisms underlying those differences. Accordingly, the interpretation of the superiority of delayed AI support was based primarily on learners’ reading comprehension performance rather than on direct measures of cognitive processes such as cognitive effort, cognitive load, inferential engagement, comprehension monitoring, attention allocation, or processing depth during reading. In addition, although the reading comprehension instrument demonstrated satisfactory psychometric properties based on complementary CTT and IRT analyses, the assessment consisted of a 10-item multiple-choice test and therefore may not have captured the full complexity of reading comprehension as a multidimensional construct. Consequently, the explanations advanced in the present study should be interpreted as theoretically informed accounts rather than empirically validated mechanisms. Future research should employ more comprehensive reading comprehension assessments incorporating a larger number of items and multiple assessment formats while also integrating process-oriented indicators such as reading time, think-aloud protocols, eye-tracking, screen recording, interaction log analysis, and validated measures of cognitive load and metacognitive processing. Such approaches would provide a more comprehensive understanding of both reading outcomes and the cognitive processes underlying AI-assisted reading.

Second, the study focused primarily on reading comprehension outcomes without examining how learners interacted with the AI system itself throughout the intervention period. Although participants completed all activities under classroom supervision and followed standardised AI-use procedures, no interaction logs or detailed records of participants’ prompt histories, prompting strategies, AI usage frequency, interaction duration, or AI-generated responses were recorded or analysed. Consequently, it was not possible to examine variation in individual AI usage behaviour or determine the extent to which such differences may have contributed to the observed reading comprehension outcomes. Accordingly, the findings should be interpreted as reflecting differences between the assigned instructional conditions rather than controlled differences in individual AI interaction behaviour. Future research could investigate prompting behaviour, the frequency of AI consultation, response-evaluation patterns, levels of dependence on AI-generated clarification during reading tasks, and treatment fidelity through the use of interaction logs, usage analytics, prompt histories, and screen-recording methods.

Third, the experimental conditions were implemented using a single AI-supported instructional configuration operating under fixed task procedures. Replication across different AI systems, reading genres, prompting structures, and instructional contexts remains necessary to determine the extent to which the present findings generalise beyond the instructional setting examined in this study. Fourth, the findings reflected immediate post-intervention performance and did not examine whether the observed advantages associated with delayed AI support would remain stable over delayed retention periods or longer-term reading development. Given that the present study evaluated immediate learning outcomes rather than the durability of the underlying learning processes, retention-based investigations remain necessary before stronger conclusions can be drawn regarding the long-term educational value of delayed AI support. Finally, although descriptive variation was observed across the literal, inferential, and global comprehension dimensions, the inferential analyses focused primarily on overall reading comprehension performance. Future research could examine these dimensions independently in order to determine whether the timing of AI support influences specific levels of textual processing differently.

## 8. Conclusions

The present study examined how the timing of AI-generated support affected EFL learners’ reading comprehension performance across three instructional conditions: delayed, immediate, and no support. Statistically significant differences were observed across the three groups, with delayed AI support producing the strongest post-test reading comprehension performance, followed by immediate support and unsupported reading. The findings suggest that the instructional value of AI-assisted reading was closely related to the stage at which explanatory assistance became available during comprehension. Although the present study did not directly examine the cognitive mechanisms underlying these differences, delayed AI support was associated with higher reading comprehension performance than both immediate support and unsupported reading under the experimental conditions. Immediate support also produced better outcomes than unsupported reading. These findings indicate that the timing of AI support represents an important instructional consideration when designing AI-assisted reading activities for EFL learners.

## Figures and Tables

**Table 1:** Item difficulty based on CTT.

Item	Difficulty	Std Dev	Std Error	Point Biserial	Drop Alpha
Lit1	0.826	0.381	0.029	0.412	0.746
Lit2	0.791	0.408	0.031	0.384	0.751
Lit3	0.628	0.485	0.037	0.429	0.742
Lit4	0.512	0.501	0.038	0.493	0.734
Inf1	0.541	0.500	0.038	0.501	0.731
Inf2	0.419	0.495	0.038	0.376	0.754
Inf3	0.430	0.497	0.038	0.489	0.735
Glo1	0.337	0.474	0.036	0.407	0.747
Glo2	0.314	0.465	0.035	0.398	0.749
Glo3	0.291	0.455	0.035	0.382	0.752

Note. Overall reliability, Cronbach’s α = 0.762.

**Table 2:** Item difficulty based on Item Response Theory.

Item	Difficulty	SE	CI Lower	CI Upper	Outfit MSQ	Infit MSQ	Discrim
Lit1	−1.481	0.084	−1.645	−1.317	0.704	0.899	1.412
Lit2	−1.625	0.084	−1.790	−1.461	0.873	0.944	0.967
Lit3	−0.588	0.084	−0.752	−0.423	0.864	0.882	1.107
Lit5	−0.036	0.084	−0.200	0.128	0.702	0.816	1.562
Inf1	−0.144	0.084	−0.308	0.020	0.707	0.811	1.575
Inf2	1.068	0.084	0.904	1.232	0.951	0.965	0.742
Inf3	0.261	0.084	0.097	0.425	0.708	0.786	1.654
Glo1	1.111	0.084	0.947	1.276	0.796	0.912	1.137
Glo2	1.169	0.084	1.005	1.333	0.830	0.911	1.148
Glo3	1.407	0.084	1.242	1.571	0.809	0.915	1.195

**Table 3:** Preliminary Tests of ANCOVA Assumptions.

Test	Statistic	df/*N*	*p*	Decision
Residual normality (Shapiro–Wilk)	0.989	90	.673	Supported
Levene’s test	0.091	2, 87	.914	Supported
Quadratic deviation after equal slopes	0.741	1, 85	.392	Not significant
Condition × pre-test interaction	0.440	2, 84	.646	Not significant

**Table 4:** Descriptive statistics for reading comprehension by instructional condition.

Instructional Condition	*N*	Pre-Test		Post-Test	
		Mean	*SD*	Mean	*SD*
Immediate Support	30	7.19	1.01	7.51	1.16
Delayed Support	30	7.21	1.05	8.43	1.14
No Support	30	7.08	1.04	6.32	1.11

**Table 5:** Tests of between-subjects effects for post-test reading comprehension.

Source	Type III *SS*	*df*	*MS*	*F*	*p*	Partial η^2^
Corrected model	83.263	3	27.754	24.911	<.001	0.465
Intercept	4958.341	1	4958.341	4450.322	<.001	0.981
Pre-test reading comprehension	16.345	1	16.345	14.670	<.001	0.146
Instructional condition	63.203	2	31.601	28.364	<.001	0.397
Error	95.817	86	1.114			

Note. Dependent variable: post-test reading comprehension. R^2^ = 0.465; adjusted R^2^ = 0.446.

**Table 6:** Estimated marginal means of post-test reading comprehension.

Instructional Condition	Adjusted Mean	SE	95% CI
Immediate Support	7.500	0.193	[7.117, 7.883]
Delayed Support	8.409	0.193	[8.026, 8.792]
No Support	6.358	0.193	[5.974, 6.741]

Note. Estimated marginal means were evaluated at the mean pre-test score of 7.158.

**Table 7:** Pairwise comparisons of adjusted post-test reading comprehension means.

(I) Instructional Condition	(J) Instructional Condition	Mean Difference (I − J)	*SE*	Bonferroni *p*	95% CI
Immediate Support	Delayed Support	−0.909	0.273	.004	[−1.574, −0.243]
Immediate Support	No Support	1.142	0.273	<.001	[0.476, 1.809]
Delayed Support	No Support	2.051	0.273	<.001	[1.385, 2.718]

Note. Comparisons are based on estimated marginal means. Bonferroni adjustment was applied to the three comparisons.

## Data Availability

The datasets generated and analysed during the current study are available from the corresponding author upon reasonable request.

## Supplementary Materials

The following supporting information can be downloaded at: https://www.mdpi.com/article/10.3390/jintelligence14090199/s1. The reading comprehension pre-test and post-test materials used in this study are available in the Supplementary Materials accompanying this article.
