# Supplementary material for: The Effects of Immediate Versus Delayed Timing of AI Support on EFL Learners’ Reading Comprehension Performance: An Experimental Study
Source: J Intell. 2026 Sep 1;14(9):199. doi: 10.3390/jintelligence14090199 (PMC13608841; doi:10.3390/jintelligence14090199)
Supplement: Supplementary file 1 [file jintelligence-14-00199-s001.zip › jintelligence-4405284-supplementary.pdf]

Reading Comprehension Pre-Test
Reading Passage
Urban Noise and Cognitive Performance

Urban environments expose individuals to a wide range of environmental stimuli, among which noise is one of the most persistent. Traffic, construction activities, and densely populated public spaces contribute to continuous background sound in many cities. Although people often adapt to these conditions, researchers have increasingly investigated how chronic exposure to urban noise may influence cognitive functioning and learning.

Noise can interfere with the brain's ability to process information efficiently. Cognitive psychologists explain that complex mental tasks require sustained attention and working memory. When background noise is present, individuals must allocate additional mental resources to filter out irrelevant auditory stimuli. This process can reduce the cognitive capacity available for the primary task, particularly when the task requires comprehension, reasoning, or memory.

Research conducted in educational contexts provides evidence for these effects. Studies comparing students working in quiet classrooms with those exposed to moderate environmental noise have shown differences in reading comprehension and memory performance. Students exposed to noise tend to make more comprehension errors and require more time to complete reading tasks. These findings suggest that environmental conditions may influence academic performance in subtle but measurable ways.

However, the relationship between noise and cognition is not entirely straightforward. Some researchers note that the impact of noise may depend on its characteristics. For example, unpredictable or fluctuating sounds appear to be more disruptive than steady background noise. Similarly, tasks that involve language processing may be particularly sensitive to auditory interference because they require precise interpretation of linguistic information.

Despite these findings, urban noise is difficult to eliminate completely. For this reason, urban planners and educators have begun exploring strategies to reduce its effects. Architectural solutions, such as sound-absorbing materials and improved building insulation, can decrease indoor noise levels. In addition, some schools implement quiet study zones designed to minimize auditory distractions during learning activities.

Understanding how environmental noise influences cognitive performance remains an important area of research. As urban populations continue to grow, identifying ways to create learning environments that support concentration and comprehension may contribute to improved educational outcomes.

Comprehension Questions
Literal Comprehension

1. What are the main sources of urban noise mentioned in the passage?
   A. Television and music
   B. Traffic and construction activities
   C. Natural sounds such as wind
   D. Household appliances
2. According to the passage, which cognitive functions are required for complex mental tasks?
   A. Attention and working memory
   B. Visual perception and creativity
   C. Motor coordination and balance
   D. Emotional awareness and empathy
3. What difference was observed between students working in quiet classrooms and those exposed to noise?
   A. No measurable difference was observed
   B. Students in noisy environments showed lower comprehension performance
   C. Students in noisy environments finished tasks faster
   D. Students in quiet classrooms showed lower motivation
4. Which strategy is suggested to reduce the effects of noise in learning environments?
   A. Increasing classroom size
   B. Using sound-absorbing materials in buildings
   C. Allowing constant background music
   D. Reducing reading activities

Inferential Comprehension

5. Why might background noise reduce cognitive performance?
   A. It requires additional mental effort to ignore irrelevant sounds
   B. It improves attention to details
   C. It increases reading speed
   D. It reduces the need for memory
6. What can be inferred about unpredictable sounds?
   A. They are less disruptive than steady noise
   B. They may interfere more strongly with cognitive tasks
   C. They improve concentration during reading
   D. They have no measurable effect on learning
7. Why are language-related tasks especially sensitive to noise?
   A. They depend on accurate interpretation of linguistic information
   B. They require physical movement
   C. They involve visual processing only
   D. They do not require attention

Global Comprehension

8. What is the primary focus of the passage?
   A. The causes of urban population growth
   B. The effects of environmental noise on cognitive functioning

C. The history of urban architecture
D. The economic impact of construction projects

9. What conclusion can be drawn from the passage?
   A. Environmental conditions may influence learning performance
   B. Noise has no impact on reading comprehension
   C. Students perform better in noisy environments
   D. Noise only affects physical health
10. Which statement best summarizes the author’s argument?
    A. Urban noise is unavoidable and therefore unimportant
    B. Environmental noise can affect cognitive processes, and strategies are needed to reduce its impact
    C. Students should study only in outdoor environments
    D. Noise mainly influences physical activity rather than cognition

Post-Test Reading Passage
Plastic Waste and Marine Ecosystems

Plastic waste has become a persistent feature of many marine environments. Large quantities of discarded plastic enter oceans each year through rivers, coastal settlements, shipping activities, and fishing operations. Unlike many natural materials, plastic decomposes very slowly. As a result, waste entering the ocean today may persist in the environment for decades, creating long-term challenges for marine ecosystems. Researchers estimate that millions of tonnes of plastic enter marine environments annually, making plastic pollution one of the most widely discussed environmental issues of recent decades.

Marine organisms are affected by plastic pollution in several ways. Larger items such as fishing nets, ropes, and plastic packaging can entangle marine animals and restrict their movement. Sea turtles, seabirds, and marine mammals are particularly vulnerable because entanglement may interfere with feeding, migration, and reproduction. Plastic fragments may also be mistaken for food. Numerous studies have documented plastic ingestion among fish, seabirds, and other marine species. Once ingested, plastic can occupy space in the digestive system without providing nutritional value, which may contribute to reduced feeding efficiency and poorer physical condition.

Recent attention has focused on microplastics, which are small plastic particles produced through the gradual breakdown of larger plastic items. Because of their size, these particles can be consumed by plankton, shellfish, and other organisms at the base of marine food webs. Scientists are concerned about this process because organisms at lower trophic levels are consumed by larger species. Consequently, plastic particles may move through marine food chains and become distributed across different levels of marine ecosystems. Although researchers continue to investigate the long-term consequences of microplastic exposure, existing evidence suggests that these particles are now present in many ocean regions worldwide.

The effects of plastic pollution are not limited to wildlife alone. Marine ecosystems support fisheries, tourism, and other economic activities that provide income for millions of people. When pollution damages coastal habitats or reduces marine biodiversity, local communities may also experience economic consequences. For this reason, environmental researchers increasingly view plastic pollution as both an ecological and socioeconomic challenge rather than a purely environmental issue.

Addressing plastic pollution presents considerable difficulties. Cleanup initiatives can remove some waste from beaches and coastal waters, but preventing pollution at its source is often regarded as a more effective strategy. Many governments have introduced policies aimed at reducing the consumption of single-use plastics and improving waste-management systems. Environmental organisations have also promoted public awareness campaigns designed to encourage more sustainable patterns of consumption and disposal.

Despite these efforts, plastic pollution remains a global concern. Marine ecosystems perform important ecological functions and support a wide range of species and human activities. Understanding the causes, consequences, and possible solutions to plastic pollution, therefore,

remains an important priority for researchers, policymakers, and environmental organisations seeking to protect ocean environments for future generations.

Comprehension Questions
Literal Comprehension

1. According to the passage, which activities contribute to plastic waste entering the oceans?
A. Rivers, coastal settlements, shipping activities, and fishing operations
B. Volcanic eruptions and earthquakes
C. Agricultural irrigation systems only
D. Natural ocean currents alone

2. What may happen when marine animals become entangled in plastic debris?
A. Their movement and feeding may be restricted
B. Their reproductive success automatically increases
C. Their access to food becomes easier
D. Their migration routes become shorter

3. What are microplastics according to the passage?
A. Small marine organisms that consume waste materials
B. Plastic particles formed through the breakdown of larger plastic items
C. Chemicals used in waste-management systems
D. Naturally occurring particles found in seawater

4. Which strategy is described in the passage as helping to reduce plastic pollution?
A. Improving waste-management systems and reducing single-use plastics
B. Expanding the use of disposable plastic packaging
C. Increasing industrial discharge into coastal waters
D. Reducing environmental monitoring programmes

Inferential Comprehension

5. Why might plastic pollution continue to affect marine ecosystems even after larger plastic items begin to break down?
A. Because the resulting microplastics can enter marine food chains and spread through different levels of the ecosystem
B. Because larger plastic items immediately disappear from the environment
C. Because marine organisms are unable to consume small plastic particles
D. Because microplastics remain concentrated in a single location

6. What can be inferred about microplastics in marine ecosystems?
A. Their effects are limited to organisms living near coastlines
B. They may spread widely because they can be transferred through marine food chains
C. They are easily removed through natural ecological processes
D. They affect only large marine mammals

7. Why do researchers view plastic pollution as more than an environmental issue?
A. It influences only scientific research activities
B. It affects marine ecosystems that support economic activities and local communities
C. It is mainly a problem for industrial manufacturers
D. It has reduced the importance of marine biodiversity

Global Comprehension

8. What is the primary focus of the passage?
A. The development of modern waste-management technologies
B. The causes, consequences, and responses to plastic pollution in marine ecosystems
C. The economic benefits of marine tourism
D. The history of environmental legislation

9. What broader message does the passage convey about addressing plastic pollution?
A. Effective responses require sustained efforts aimed at both reducing waste and protecting marine ecosystems
B. Plastic pollution can be eliminated entirely through beach clean-up campaigns
C. Marine ecosystems are largely unaffected by human waste-management practices
D. The responsibility for addressing plastic pollution belongs only to environmental organisations

10. Which statement best summarises the author's argument?
A. Plastic pollution is an unavoidable consequence of economic development
B. Marine pollution affects only coastal regions and wildlife populations
C. Plastic pollution presents long-term challenges for marine ecosystems and requires coordinated efforts to reduce its impacts
D. Environmental organisations are solely responsible for addressing marine pollution
